# Probing Buried Interfaces in Batteries: Toward *Operando* Visibility and Quantitative Diagnosis

**DOI:** 10.1021/acs.chemmater.5c03241

**Published:** 2026-02-16

**Authors:** Zhao Li, Aigerim Omirkhan, Christopher Nicklin, Mary P. Ryan

**Affiliations:** † Department of Materials, Royal School of Mines, 4615 Imperial College London, South Kensington Campus, SW7 2AZ London, U.K.; ‡ Diamond Light Source, Rutherford Appleton Laboratory, OX11 0DE Didcot, U.K.

## Abstract

The evolution of buried interfaces, the hidden junctions where distinct phases exchange charge, mass, and mechanical response under nonequilibrium conditions, strongly influences the performance and stability of functional devices such as batteries, but they remain difficult to probe directly. This perspective summarizes the types of buried interfaces that form within battery electrodes and their electrochemical function in the device, and it discusses how advances in *operando* probes, cell architectures, and multimodal and correlative strategies have enabled dynamic and chemically specific visibility of their evolution. Despite this progress, *operando* signals remain challenging to interpret because they are affected by, for example, beam damage-induced changes, variations in *operando* cell geometry, and intrinsic sample-to-sample differences, which together limit quantitative insight. Building on these considerations, the perspective examines how *operando* visibility can be transformed into quantitative diagnosis by integrating multimodal measurements with physically informed interface models and data-driven analysis. The final section outlines a roadmap for reproducible and quantitative *operando* analysis, centered on standardized cell architectures, long-term autonomous measurements, and artificial intelligence approaches that incorporate physical constraints. In summary, these developments define a pathway from *operando* visibility to quantitative diagnosis and provide a foundation for advancing interface characterization and quantitative analysis in batteries and related energy materials.

## Graphical abstract

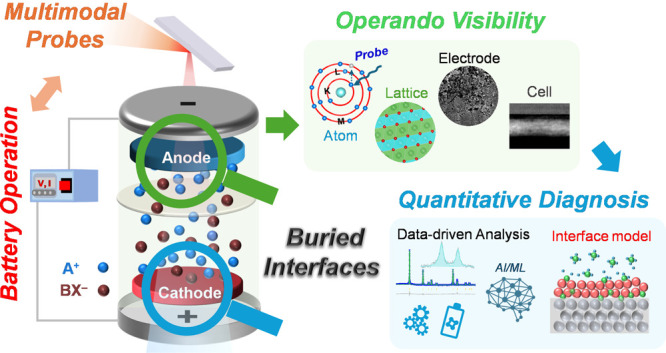

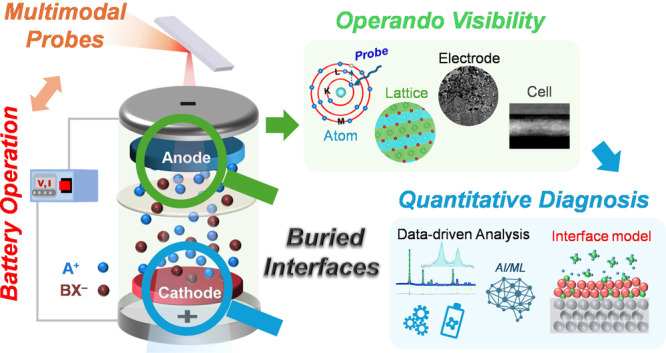

## 1 Introduction

The performance and reliability of rechargeable batteries are governed by buried interfaces, the hidden junctions where distinct phases exchange charge, mass, and chemomechanics under nonequilibrium conditions.^1–6^ These regions form a hierarchically coupled network rather than isolated boundaries, and their collective evolution determines electrochemical efficiency, stability, and lifetime.^3–5,7^ Understanding their transformation under realistic operating conditions is central to advancing both fundamental interface science and practical energy materials design.^7–9^

Conventional characterization has advanced interfacial chemistry, yet *ex situ* measurements are experimentally convenient but often distort interfacial states after disassembly.^5,7,10–12^ *In situ* techniques preserve the electrolyte environment but are typically performed under steady state or weakly perturbed electrochemical conditions, often relying on simplified geometries or half-cell configurations that do not fully represent realistic device operation.^7,8,10–15^ *Operando* characterization overcomes these limitations by probing intact cells directly, under applied current or potential control, capturing the coupled chemical, mechanical, and transport processes that define realistic interfacial behavior.^7,11,12,16–19^ This emergence of *operando* visibility, defined as dynamic, nondestructive, and chemically specific observation of buried interfaces, has transformed these regions from conceptual abstractions into experimentally accessible systems.^11,20–23^

Recent progress in high-brilliance synchrotron and neutron sources, radiation-tolerant *operando* cells and correlative analytical workflows has extended the depth, temporal resolution and multimodal reach of *operando* visibility.^12,19,21,22,24–29^ X-ray, neutron, optical, electron, and magnetic resonance probes now offer complementary contrasts spanning atomic to device scales, enabling real-time tracking of structural, chemical, and mechanical evolution within working electrodes.^16,22,27,28,30–39^ When integrated, these approaches link molecular interactions with mesoscale architectures and macroscopic degradation, providing an unprecedented picture of buried interface evolution.^9,21,26,40,41^

Yet, “visibility” alone does not yield quantitative understanding.^11,19,29^ *Operando* signals arise from overlapping chemical, structural, and mechanical processes; sampling differences across probes limit multimodal alignment; and beam damage together with cell geometries that diverge from practical electrodes can bias the observed interfacial evolution.^11,19,26,29,42–44^

Achieving quantitative diagnosis therefore requires models that are grounded in physics, standardized and reproducible *operando* platforms, and data-driven methods capable of extracting mechanistic descriptors from coupled multiscale evolution.^3,40,45–48^

Most existing reviews focus on specific *operando* techniques or on general *in situ* and *operando* concepts, whereas a unified perspective that connects buried interfaces hierarchy, multimodal visibility, and quantitative interpretation has been lacking. Within this context, this perspective examines the transition from *operando* visibility to quantitative diagnosis of buried interfaces. Section 2 outlines the hierarchy of buried interfaces and their electrochemical functions. Section 3 discusses multimodal probes and correlative strategies that establish *operando* interrogation and visibility. Section 4 introduces a conceptual and practical framework for translating these observations into a quantitative and reproducible interface diagnosis. Together, these elements define a pathway from visualizing interfacial evolution to diagnosing the mechanisms that govern electrochemical function and degradation.

## 2 Types of Buried Interfaces and Their Electrochemical Function

Buried interfaces define the internal architecture through which ions, electrons, and mechanical stress are transmitted in rechargeable batteries. Rather than idealized planar boundaries, they form a hierarchically interconnected network that permeates electrodes and current collectors and reorganizes during operation.^1,2^ Understanding this dynamic coupling is essential for bridging *operando* visualization with quantitative interface science. Figure 1 schematically illustrates this hierarchy, from molecular-scale electrode–electrolyte boundaries in an idealized (pristine) battery to the complex buried interfaces that develop within composite electrodes over time.

**Figure 1:**
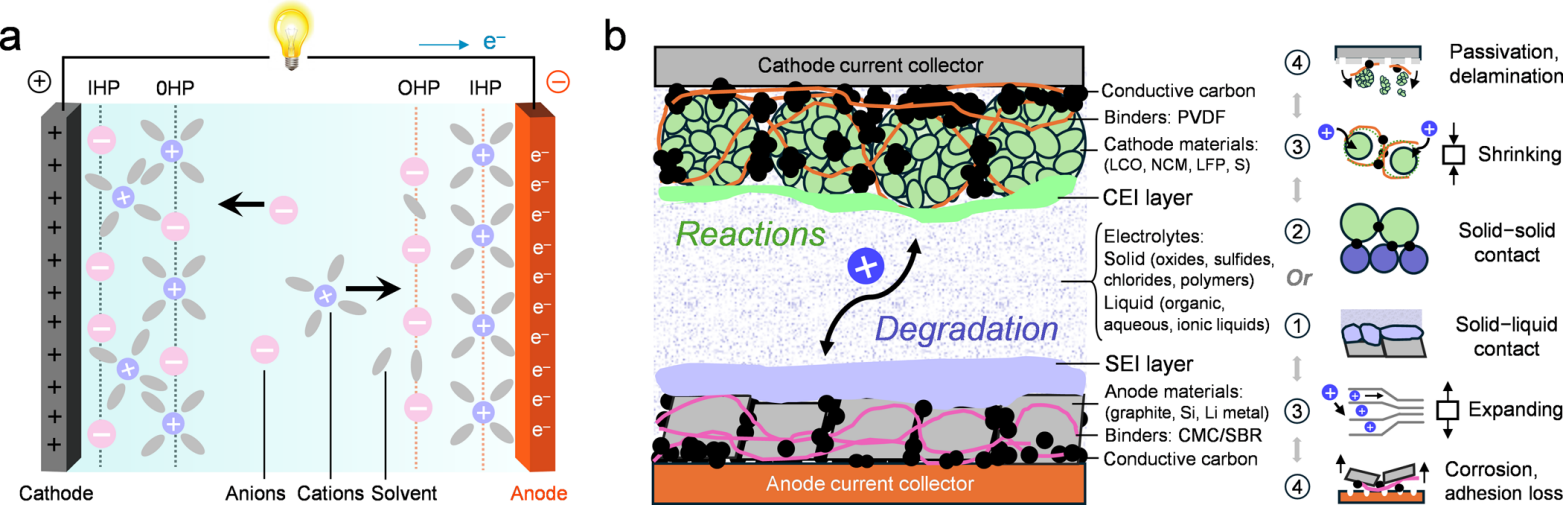
Schematic representation of buried interfaces in rechargeable batteries. (a) Formation of electrical double layers at solid–liquid boundaries during charging, illustrating the spatial organization of solvated ions and solvent molecules that precedes interphase nucleation. (b) Extension to composite electrodes, mapping the four classes of buried interfaces across solid–liquid or solid–solid contacts among active materials, electrolytes, binders, and current collectors. Chemomechanically driven reactions and degradation processes couple these interfaces across multiscales, forming a hierarchically interconnected network that governs performance and failure.

In rechargeable batteries, these interfaces can be broadly classified into four interrelated types: (1) solid–liquid boundaries where the solid electrolyte interphase (SEI) and cathode electrolyte interphase (CEI) form; (2) solid–solid contacts in all-solid-state configurations; (3) mesoscale interparticle and binder interfaces within composite electrodes; and (4) buried contacts between the electrode composite and the current collector.^1–4,8,14^ Although distinct in chemistry and morphology, these interfaces are mechanically and electronically coupled, so that local instabilities propagate throughout the electrode architecture.^4,6,40^ Among them, solid–liquid interfaces have been studied most extensively, as they exemplify how electrochemical and mechanical processes intertwine at buried boundaries.^2,8,35^

### 2.1 Solid–Liquid Electrochemical Interfaces: From Solvation Dynamics to Interphase Formation

At solid–liquid boundaries, the arrangement of solvated ions and solvent molecules under applied potential defines the precursor molecular structure from which interphases emerge. The electrical double layer establishes the reactive environment where electrolyte reduction and oxidation generate nanoscale interphases such as the SEI and CEI.^1–3,14^ These interphases are chemically heterogeneous and continuously reconstruct during cycling, mediating both ion transport and surface passivation.^1–4^ Their nonequilibrium evolution in density, composition, and stress strongly influences efficiency and lifetime yet remains largely inaccessible to conventional characterization. Recent *operando* measurements using complementary probes have begun to capture these transformations in real time, revealing quantitative correlations between interfacial chemistry and electrochemical performance.^8,9,19,21,35,39^

### 2.2 Solid–Solid Interfaces in Solid-State Batteries: Interfacial Reactions and Ionic Bottlenecks

In solid-state batteries, charge transport occurs across compact solid–solid contacts rather than through liquid electrolytes. These interfaces frequently undergo redox-driven reactions that form resistive layers or voids, creating ionic bottlenecks and strain heterogeneity that limit rate capability and mechanical integrity.^4,8,27^ Even nominally stable materials can decompose electrochemically, generating localized stress and delamination. Quantitative *operando* methods that combine scattering, spectroscopy, and modeling are required to resolve these interfacial reactions and to link local chemistry and stress evolution with macroscopic degradation and impedance behavior.

### 2.3 Interparticle and Binder Interfaces: Mesoscale Coupling of Transport and Chemomechanics

Within composite electrodes, mesoscale networks of interparticle and binder interfaces connect active materials, conductive additives, and current collectors. These buried junctions govern percolation, stress distribution, and mechanical cohesion but reorganize continuously during cycling.^6,26,40^ Variations in the contact area, binder degradation, or particle rearrangement disrupt percolation pathways and create spatially uneven electronic and ionic conductivity. These changes generate locally concentrated current pathways and associated overpotential gradients, which in turn drive heterogeneous strain accumulation and macroscopic degradation.^6,49^ *Operando* imaging and mechanical mapping have demonstrated that these buried interfaces dynamically exchange stress and charge, providing crucial insight into electrode-level fatigue and failure mechanisms.^6,41,49,50^

### 2.4 Current Collector Interfaces: Electrochemomechanical Coupling and Interfacial Stability

The interface between the composite electrode and the current collector, although deeply buried, is critical for both electrical conduction and mechanical stability.^6,51,52^ Electrochemical reactions, oxide growth, and incipient delamination at this boundary increase the interfacial resistance and progressively undermine the electrical contact quality. Experimental measurements on lithium-ion battery assemblies show that electrical contact resistance at this interface can contribute up to about 20% of the total energy flow under normal operating conditions, with losses substantially reduced only when sufficient clamping/calendering pressure or interfacial conductive media improve the real contact area.^53^ *Operando* scattering, acoustic, and tomographic methods now enable visualization of adhesion loss, oxide growth, and stress evolution, offering a route to quantify electrochemomechanical coupling at this essential buried contact.^51,54^

### 2.5 Hierarchical Coupling across Buried Interfaces: From Local Reactions to Collective Degradation

All buried interfaces are interconnected within a hierarchical network that couples electrochemical reactions with mechanical feedback.^2,4,5,9^ Local redox events or strain accumulation at one boundary can cascade through mesoscale networks, blurring distinctions between chemical instability and mechanical fatigue. *Operando* measurements in practical electrodes, even those obtained with high spatial resolution techniques, almost always contain convoluted signals originating from several interfaces at once.^29,40^ This inherent overlap makes the separation of coupled interfacial dynamics a central challenge and defines the frontier between *operando* visibility and quantitative diagnosis. Addressing this challenge requires experimental approaches capable of tracking buried interfaces under realistic operating conditions, which is the focus of Section 3.

## 3 *Operando* Visibility of Buried Interfaces: Probes and Multimodal Approaches

Buried interfaces play central roles in electrochemical and mechanical response, yet their evolution has long remained inaccessible to direct observation.^5,17,55^ The emergence of *operando* visibility marks a conceptual transition in interface science.^7,8,10,17,19^ It transforms degradation studies from inference to direct measurement, linking molecular interactions with device-level behavior. Figure 2a summarizes the progression from *ex situ* and *in situ* approaches to full *operando* methodologies, highlighting how the multiscale visibility of buried interfaces can be achieved through complementary probes. This progression from static to dynamic measurement motivates the multimodal *operando* strategies discussed later.

**Figure 2:**
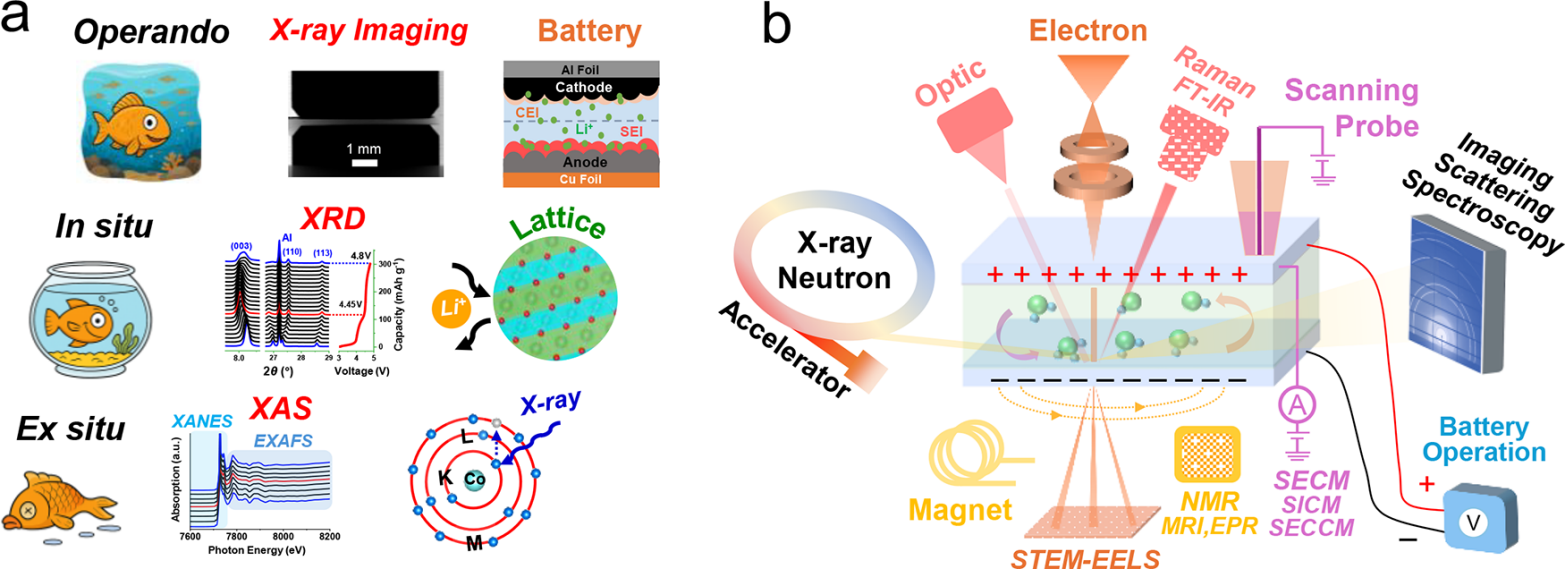
From *ex situ* to *operando* characterization and multimodal probe visibility of buried interfaces. (a) Conceptual evolution from *ex situ* and *in situ* to *operando* characterization. Only *operando* methods capture the dynamic behavior of electrochemical systems under operating conditions, preserving their native chemical and mechanical states that would otherwise change after cell disassembly. (b) Complementary probes enabling multiscale *operando* visibility. X-ray, neutron, optical, electron, scanning probe, and magnetic resonance techniques provide distinct penetration depths and contrast mechanisms that together access buried interfaces from atomic to device scales.

### 3.1 Penetrating Probes: X-Ray and Neutron Techniques

Penetrating radiation probes provide nondestructive access to buried interfaces within electrochemically active and mechanically constrained electrodes.^17,21,28^ X-ray-based techniques span a broad hierarchy of contrast mechanisms and accessible length scales, and together they reveal how interfacial chemistry, crystallography, and nanostructure evolve during battery operation.^10,13,21,22,56,57^ Wide-angle X-ray scattering (WAXS), which includes both Bragg diffraction and diffuse scattering from partially ordered regions, captures crystallographic evolution, lattice strain, and phase transitions across full electrode thicknesses.^19,21,22,57^ Small-angle scattering (SAXS) complements this capability by resolving nanoscale density fluctuations associated with porous domains, interphase aggregates, and mesoscale structural reorganization that often precede long-range crystallographic changes.^19,21,22^

The sensitivity of X-ray measurements to buried interfacial layers is strongly governed by the incident geometry and the *operando* cell that defines the beam path, as illustrated in Figure 3. In conventional transmission and reflection configurations, the beam penetrates the full electrode thickness, and the resulting scattering is dominated by bulk contributions (Figure 3a), which limits sensitivity to interfacial regions near the electrode surface. Transmission mode measurements using open window coin cell configurations, as shown in Figure 3b, are widely employed to monitor the bulk structural evolution during electrochemical cycling. However, their ability to resolve buried interphases remains limited because the illuminated volume spans the entire electrode thickness. In contrast, grazing incidence techniques, as shown in Figure 3c, operate at incident angles close to the critical angle, which confines the X-ray penetration depth to the near-surface region of the electrode that hosts the electrolyte contacting buried interphases. This ensures that the probed “near surface” in an *operando* cell corresponds directly to buried solid–liquid interfaces rather than an exposed surface. By restricting the probed depth to the first tens to hundreds of nanometers from this interface, these geometries selectively enhance scattering from interfacial layers relative to the underlying bulk while preserving realistic electrochemical conditions.^6,12,15,22,58^

**Figure 3:**
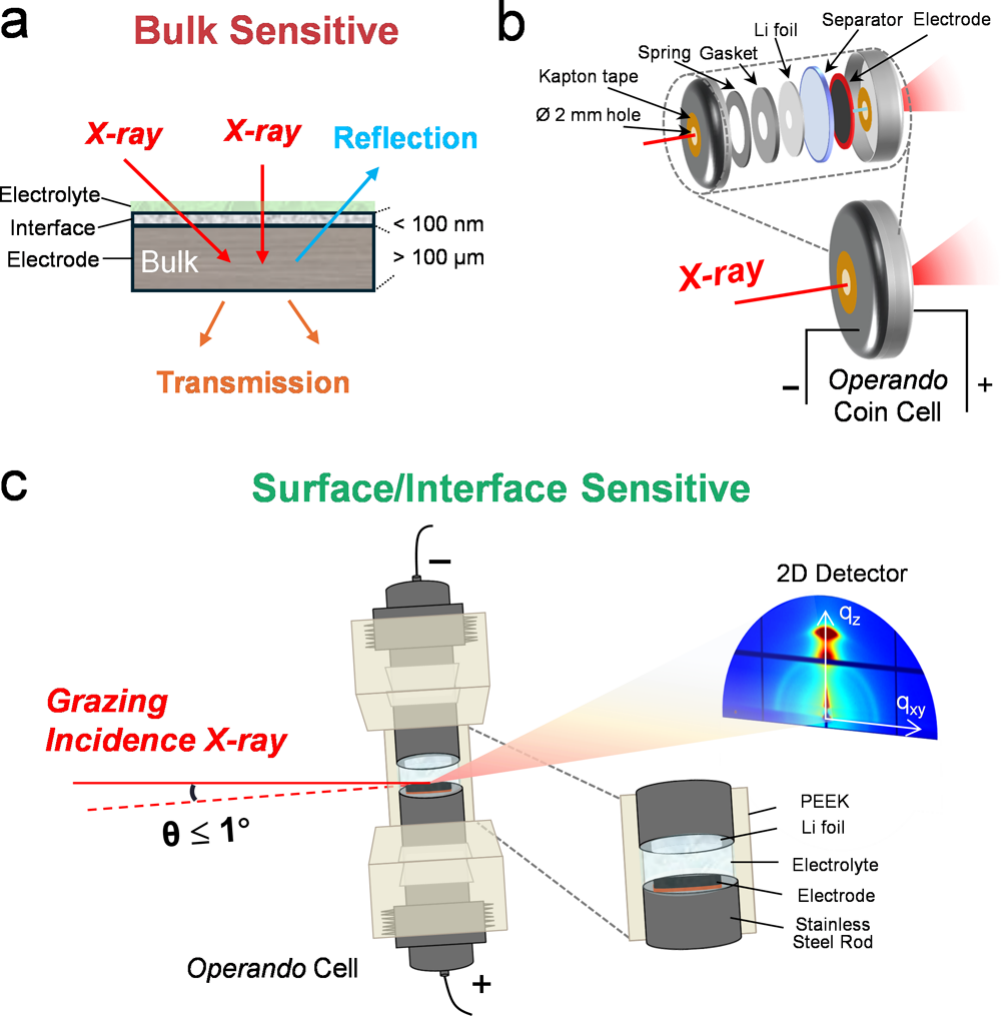
X-ray geometry and *operando* cell design for accessing buried interfaces. (a) Transmission and reflection geometries are dominated by bulk signals as the beam penetrates the full electrode, which often obscures subtle interfacial transformations. (b) *Operando* coin cells in transmission mode enable bulk structural tracking but provide limited access to buried interfaces. (c) Grazing incidence geometries restrict penetration to the interfacial near-surface region, and dedicated *operando* cell designs preserve unobstructed low angle access for interface sensitive measurements.

Grazing incidence wide-angle and small-angle X-ray scattering (GIWAXS and GISAXS) reveal interfacial strain, structural disorder, and nanoscale density variations associated with porous layers, interphase clusters, and surface-correlated heterogeneity.^22,58^ X-ray reflectivity (XRR) provides complementary subnanometer sensitivity to thickness and electron density profiles within layered interfacial structures, enabling quantitative reconstruction of early-stage interphase formation and solvent penetration.^12,22,58^ These measurements rely on *operando* cell architectures that maintain unobstructed shallow angle access and stable alignment during cycling.^8,12,22^

Spectroscopic probes add chemically specific information that is essential for the interpretation of interfacial evolution. X-ray absorption spectroscopy (XAS) and resonant inelastic X-ray scattering (RIXS) provide oxidation states, coordination environments, and electronic structure within bulk and interfacial regions.^13,37,59^ Soft X-ray absorption spectroscopy (sXAS) and X-ray photoelectron spectroscopy (XPS) access the outer nanometers of reactive interfaces, where electrolyte reduction, surface reconstruction, and initial interphase formation occur.^35,36^ Although these measurements are traditionally conducted under high vacuum, advanced implementations have enabled *in situ* and *operando* studies through the development of liquid-compatible soft X-ray cells and ambient pressure X-ray photoelectron spectroscopy (APXPS).^36,60^ These approaches now permit chemically specific probing of buried solid–liquid and solid–solid interfaces under electrochemically relevant states, complementing the bulk-sensitive insight available from hard X-ray absorption spectroscopy.

Neutron probes complement X-ray techniques through their isotopic contrast and sensitivity to light elements such as lithium, hydrogen, and oxygen.^27^ Neutron reflectometry (NR), small-angle neutron scattering (SANS), and quasi-elastic neutron scattering (QENS) enable nanoscale mapping of isotopic distributions, diffusion pathways, and density variations within buried regions.^28,38,39^ Because neutron and X-ray interactions rely on distinct scattering mechanisms, combining them links redox gradients, strain evolution, and mass transport across complex electrode architectures.^26,41^ Together, these penetrating probes provide quantitative access to buried interfacial processes while maintaining realistic electrochemical conditions.

### 3.2 Local Probes: Optical, Electron, and Scanning Probe Techniques

While X-ray and neutron probes capture average structural and chemical evolution across buried interfaces, local probes, including optical, electron, and scanning techniques, provide spatially resolved insights into morphology, chemistry, and structure at specific sites.^8,16,33^ Vibrational spectroscopies, including confocal Raman spectroscopy and Fourier transform infrared spectroscopy (FTIR), detect transient intermediates and solvent configurations at reactive interfaces, providing molecular-level insight into electrolyte reduction, interphase nucleation, and solvent orientation under bias.^30,61,62^

Electron microscopy enables atomic-scale visualization of structural and chemical evolution at buried interfaces. *In situ* and *operando* transmission electron microscopy (TEM) and scanning transmission electron microscopy (STEM), including liquid cell configurations and four-dimensional modalities (4D-STEM) that record position-resolved diffraction patterns, capture interphase formation, gas evolution, and localized deformation within electrochemical environments.^33^ When coupled with electron energy loss spectroscopy (EELS) or energy-dispersive X-ray spectroscopy (EDX), these methods quantitatively correlate bonding, redox evolution, and structural transformation.

Scanning probe techniques enable the spatially resolved characterization of interfacial heterogeneity. Scanning electrochemical microscopy (SECM) maps local electrochemical activity through diffusion-controlled feedback with an ultramicroelectrode.^23,63^ Complementarily, scanning ion conductance microscopy (SICM) monitors interfacial topography and ionic transport using an electrolyte-filled nanopipette.^64^ By contrast, scanning electrochemical cell microscopy (SECCM) enables direct, site-specific electrochemical measurements by confining the electrolyte to a nanoscale meniscus cell,^65^ while atomic force microscopy (AFM) remains a benchmark for probing nanoscale morphology and mechanics.^49^

### 3.3 Atomic and Ionic Probes: Magnetic Resonance Techniques

Magnetic resonance techniques leverage nuclear and electron spins to provide element-specific and nondestructive insights into atomic coordination and ionic transport environments at battery interfaces. Nuclear magnetic resonance (NMR), magnetic resonance imaging (MRI), and electron paramagnetic resonance (EPR) contribute complementary *operando* capabilities.^66–71^ *Operando* NMR and MRI track chemical evolution and spatial distribution of species across electrodes and electrolytes,^70,71^ while pulsed field gradient NMR yields diffusion coefficients and identifies confinement effects and grain boundary barriers that are inaccessible to scattering or microscopy.^68^ Solid-state NMR resolves ion exchange and local structural relaxation at solid–solid contacts, linking interfacial transport to chemomechanical stability.^66,68,72^ EPR detects unpaired electron species that mediate charge transfer and degradation, revealing transient radicals, paramagnetic centers, and defect sites that control kinetics within buried regions.^73–75^ Magnetic resonance therefore bridges atomic-scale coordination with mesoscale transport and electronic reactivity, offering a radiation-free and quantitatively interpretable view of buried interface dynamics.

### 3.4 Correlative and Multimodal *Operando* Strategies

No single probe can capture the entire spectrum of coupled electrochemical, mechanical, and transport phenomena at buried interfaces.^7,9,18,24^ Comprehensive understanding requires correlative and multimodal approaches that integrate complementary contrasts within unified chemomechanical frameworks.^9,19,26^ Within a single modality such as X-rays, scattering quantifies structural evolution from porosity to lattice strain and texture,^22,58^ spectroscopy maps oxidation states and coordination,^34,37,59^ and tomographic imaging visualizes crack propagation and morphological heterogeneity.^20,41,50^ Spatial and temporal registration of these data sets enables direct correlation of strain fields from diffraction with redox gradients from spectroscopy and morphological changes from imaging, providing a cross-validated picture of interfacial evolution. For conversion-type (*e.g.*, Li–S and Li–Se) and multivalent metal-ion (*e.g.*, Mg^2+^, Zn^2+^, and Al^3+^) batteries, correlative and multimodal strategies are essential to capture the coupled evolution of solid phases and transient liquid-phase intermediates, enabling key aspects of dissolution–precipitation kinetics that govern interfacial stability.^24,51,57,76^

Multimodal integration further enhances interpretability by combining complementary contrasts that extend beyond the capabilities of a single probe. Combined X-ray and neutron measurements combine heavy element sensitivity with isotopic contrast,^26,41^ and correlated X-ray and electron workflows align synchrotron data sets with TEM or STEM-EELS observations to link mesoscale strain and phase evolution with atomic-scale motifs such as amorphous SEI domains or void nucleation at solid electrolyte boundaries.^21,33,34^ Similarly, combinations of X-ray with optical or scanning probe methods permit simultaneous monitoring of electrochemical activity, mechanical response, and network connectivity within common *operando* environments.^16^ ,^19^ ,^23^ These multimodal integrations, as illustrated in Figure 2b, establish a methodological bridge between atomic mechanisms and device-scale degradation, forming the basis for quantitative diagnosis of buried interfaces.

### 3.5 Challenges in Achieving Reliable *Operando* Visibility

Multimodal *operando* techniques offer powerful means to visualize buried interfaces, yet their reliability remains constrained by fundamental limitations rooted in cell geometry, measurement configuration, and probe interactions with reactive materials.^5,22,29^ These limitations become even more pronounced when *operando* approaches move beyond controlled thin-film systems to the complex chemomechanical environments of full-scale practical battery electrodes. Figure 4 provides a conceptual overview of these limitations by mapping key experimental constraints onto their consequences for the fidelity and interpretability of *operando* interfacial measurements.

**Figure 4:**
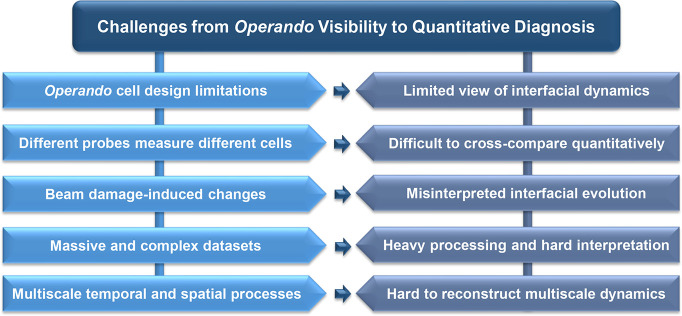
Key challenges from *operando* visibility to quantitative interfacial diagnosis. *Operando* measurements remain limited by constraints in cell architecture, by the fact that different probes require different cells and sampling geometries, and by beam damage-induced changes that can alter sensitive interphases. The massive, heterogeneous data sets generated during *operando* experiments, together with the multiscale nature of interfacial processes, yield only partial or convoluted views of interfacial evolution. These constraints collectively limit the fidelity of *operando* visibility and underscore the need for quantitative diagnostic frameworks.

The primary challenge arises from the *operando* cell design. Surface-sensitive geometries such as grazing incidence scattering, reflectometry, and photoelectron spectroscopy often require planar films, low electrolyte volumes, or simplified formulations to maintain optical access and stable alignment.^8,12,58^ These model systems differ from porous electrodes in which tortuosity, particle connectivity, electrolyte wetting, and stack pressure govern interfacial evolution, meaning that *operando*-observed interfaces may represent only a subset of those formed under commercial cycling.^21^ Moreover, the *operando* cell must be tailored to the unique physics of diverse systems. For instance, flow batteries require microfluidic designs that maintain realistic mass transport under flowing conditions, while probing interfaces in solid-state batteries requires the application of stack pressure.

The second challenge reflects the diversity of the probe requirements. Techniques, such as X-ray diffraction and spectroscopy, neutron scattering, Raman spectroscopy, optical imaging, and electron microscopy, impose distinct constraints on geometry, window materials, sample thickness, and beam paths.^16,22,33,36,37^ As a result, scattering, spectroscopy, and imaging measurements are often performed on nominally similar but not identical cells that differ in structural, chemical, and electrochemical history, with each probe sampling a different volume or depth. Variations in spatial resolution, sampling depth, and temporal cadence further complicate correlation, since features that appear as discrete voids or particles in microscopy may manifest only as averaged signatures in diffraction or spectroscopy. Integrated multimodal platforms are emerging at large-scale facilities but remain limited in availability and often require compromises in temporal or spatial resolution and electrochemical fidelity.^20,77,78^

Probe-induced interfacial changes introduce an additional source of uncertainty. High-flux X-ray beams can cause local heating, radiolytic electrolyte decomposition, or dose-dependent changes in interphase chemistry, whereas electron beams may restructure polymer-rich or beam-sensitive layers or induce charging.^42–44,79–81^ These responses, collectively termed beam damage, can generate interfacial evolution that is not representative of normal operation and can even occur under open-circuit conditions.^42,43,79,82^ Distinguishing intrinsic electrochemical evolution from beam damage-induced changes therefore requires rigorous open-circuit control experiments.^82^ Cryogenic approaches such as cryo-TEM and cryo-XPS can mitigate beam-induced artifacts by rapidly freezing beam-sensitive interphases, but they cannot currently provide *operando* measurements.^32,34,81^

The increasing complexity of *operando* data sets presents further difficulty. Spatially resolved scattering, energy-dependent spectroscopy, time-resolved imaging, and tomographic reconstructions generate high-dimensional data sets in which differences in sampling depth, probed volume, and detector noise can obscure or distort subtle interfacial changes.^29,46^ These challenges are amplified at time-limited beamlines, where decisions and adjustments must often be made on partially processed data.

*Operando* probes also capture processes across disparate time and length scales, from electronic or solvent reorganization on picosecond to nanosecond time scales to interphase growth and mechanical deformation over minutes or hours.^9,24,26^ Techniques sensitive to atomic coordination or electronic structure interrogate small sampling volumes at high temporal resolution, whereas imaging approaches access larger regions at slower cadence.^12,22,41^ Each method therefore provides only a partial view of interfacial behavior, and these views do not always overlap, producing averaged or incomplete representations of multiscale evolution.

Together, these limitations define the boundaries of the current *operando* visibility. Although modern probes offer unprecedented access to buried interfaces, holistic analysis remains constrained by cell architecture, probe interactions, and measurement geometry. Reliable visibility requires careful attention to cell representativeness, coordinated measurement strategies, rigorous control experiments, and transparent documentation of conditions, which together underpin the quantitative diagnosis developed in Section 4.

## 4 From *Operando* Visibility to Quantitative Diagnosis

*Operando* techniques have transformed buried interfaces from inaccessible regions into dynamically observable systems, yet visibility alone does not provide the physical insight required for a predictive understanding. Images, spectra, and diffraction patterns reveal how interfaces evolve, but they do not directly quantify the parameters that govern charge transfer, reaction fronts, mechanical deformation, or interphase reconstruction. For interfacial processes that control degradation and lifetime, the key question is whether *operando* measurements can be converted to reproducible mechanistic descriptors. The transition from *operando* visibility to holistic quantitative diagnosis therefore defines a central challenge for advancing electrochemical interface science, building directly on the limitations in visibility and interpretability summarized in Figure 4.

### 4.1 Challenges in Achieving Reliable Quantitative *Operando* Analysis

Limitations that constrain *operando* visibility become even more consequential when quantitative interpretation that can be consistently compared across cells, chemistries, and facilities is required. Differences in cell architecture, probe configuration, and sampling depth, together with beam-sample interactions and the complexity of high-dimensional data sets, translate directly into uncertainty in the extracted interfacial parameters.^29^ Quantitative analysis must therefore link *operando* signals to physically meaningful descriptors in a reproducible and rigorous manner.

A central challenge is that *operando* measurements are composite responses rather than direct observations of individual interfacial quantities.^11^ The measured response is a composite of coupled chemical, structural, electronic, and mechanical processes, including charge transfer, ion transport, solvent reorganization, gas evolution, and strain development, which occur concurrently across multiscales.^3,6^ Because no single mechanism dominates, calibration uncertainties, nonlinear relationships between signal and state, and the lack of stable reference states limit how precisely *operando* data can be converted into specific interfacial quantities. Quantitative diagnosis must therefore embrace the inherent multiscale and multiphysics character of interface evolution.

These difficulties intensify in multimodal experiments.^26^ Scattering, spectroscopy, imaging, and electrochemical probes interrogate different depths, volumes, and time scales, and even nominally identical cells may differ in wetting, electrolyte distribution, or mechanical confinement. As a result, data sets that appear to probe the same interface may in fact correspond to different local environments, and apparent correlations can arise from sampling differences rather than genuine coupling. Reliable interpretation requires an understanding of how each probe interacts with the evolving electrode environment and how this interaction shapes the recorded response.

Models become essential once physical parameters are extracted from the *operando* data. Any mapping between signal and interfacial state depends on assumptions about geometry, kinetics or chemistry, and small uncertainties in these assumptions can propagate into large variations in the inferred quantities.^3,48^ Rigorous quantitative analysis therefore requires transparent model selection, evaluation of parameter identifiability, and explicit assessment of underlying assumptions. Figure 5 summarizes a unified framework that links *operando* visibility, interface modeling, and data-driven interpretation into a diagnostic structure that converts qualitative observations into mechanistic insight.

**Figure 5:**
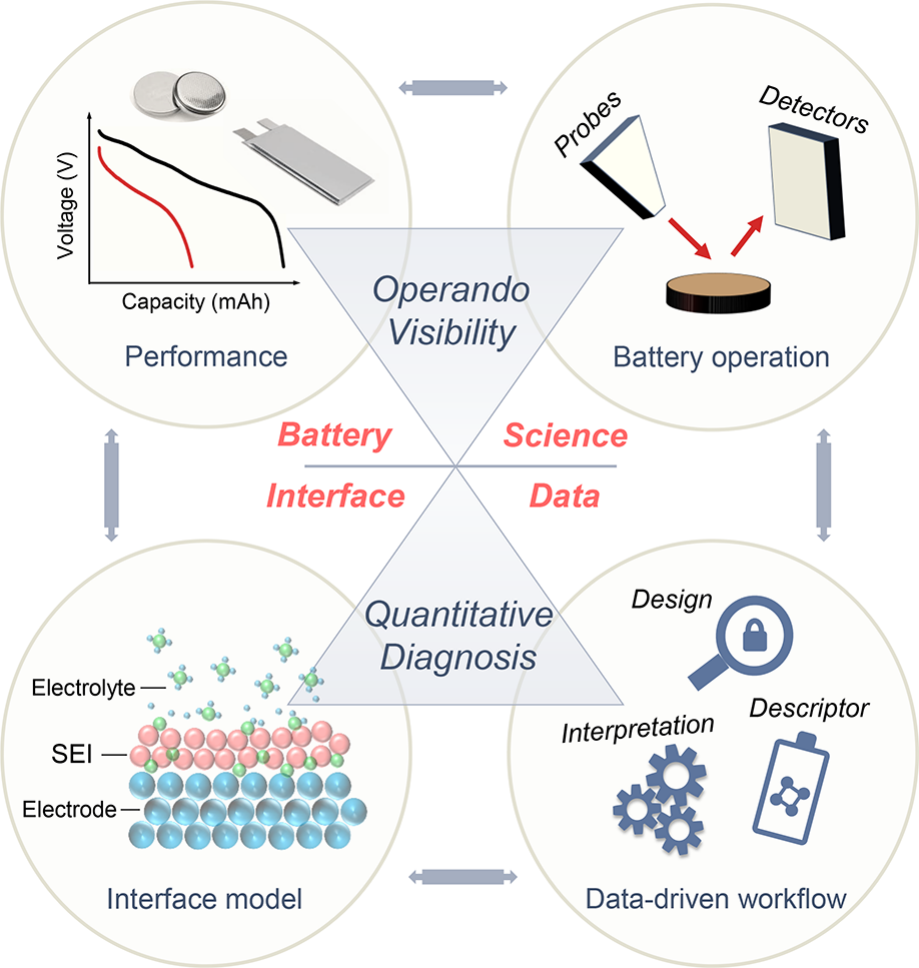
Framework linking *operando* visibility to quantitative interface diagnosis. Electrochemical degradation represents the macroscopic manifestation of interfacial evolution, while probes and detectors define measurement sensitivity and perturbation. Interface models translate buried interphases, such as the SEI, into physically interpretable constructs. Data-driven workflows integrate multimodal signals to yield descriptors coupling chemistry, mechanics, and transport, establishing an iterative framework connecting observation, measurement, modeling, and predictive understanding.

### 4.2 Unified Framework for Quantitative Interface Diagnosis

Quantitative *operando* analysis requires a framework that relates the observed evolution to the physical processes that determine interfacial function.

- *Operando* visibility provides time-resolved signatures of charge transfer, mechanical deformation, and interphase formation.^8,22^

- Interface models express these signatures as quantities such as resistivity, interphase thickness, and reaction front position or strain, enabling comparison and prediction.^3,6,11^

- Data-driven workflows integrate high-dimensional *operando* data sets into physically interpretable descriptors, defined as model-informed quantities extracted from *operando* signals that couple chemistry, mechanics, and transport, allowing consistent trends and mechanisms to emerge across diverse measurements.^40,47,83^

A defining feature of this framework is its iterative nature. Interpretation identifies the variables and regions most sensitive to interfacial evolution, refining the design of subsequent experiments. The resulting descriptors can then be directly compared with model predictions, allowing model parameters to be refined and indicate where additional *operando* measurements are needed. Through this feedback process, *operando* visibility and quantitative interpretation codevelop and sharpen mechanistic understanding and strengthen quantitative diagnosis.

Descriptors derived from this integration must be physically grounded and transferable across materials and operating conditions. When generated through physically informed modeling and multimodal *operando* results, they serve as robust markers for comparison, prediction, and materials design.^83^ The framework in Figure 5 therefore establishes the conceptual basis for converting qualitative observation into a quantitative diagnosis.

Herein, we illustrate how *operando* measurements can be converted into intuitive descriptors by using examples from interphase studies. In SEI and CEI studies, interphase formation and evolution can directly govern performance degradation. Multimodal *operando* measurements generate high-dimensional signals that require interface models to extract physically grounded descriptors. Grazing incidence scattering measurements are interpreted using layered density models to yield structural descriptors such as interphase thickness and roughness.^12,58^ Spectroscopic measurements are analyzed by reference-based spectral modeling to extract chemical descriptors such as composition or oxidation state metrics.^8,61,84^ Electrochemical impedance is interpreted using equivalent circuit models to obtain functional descriptors including interfacial resistance, polarization, and diffusion parameters.^85,86^ Imaging-based measurements provide a spatial perspective, in which image sequences are analyzed to extract descriptors of interphase coverage, heterogeneity, and porosity or crack fraction.^50,54^ As these descriptors accumulate into time-resolved, multimodal data sets, data-driven workflows integrate them into a unified descriptor space, directly linking interphase evolution to macroscopic performance degradation and enabling iterative refinement of interface models and *operando* strategies.

### 4.3 Roadmap toward Quantitative *Operando* Interface Diagnosis

Translating this framework into practice requires advances in the *operando* experimental and analytical infrastructure. Many current studies rely on customized cell designs, manually controlled long-term measurements, and labor-intensive data interpretation, limiting reproducibility and constraining quantitative comparison. Figure 6 outlines a practical roadmap that addresses these limitations through standardization, autonomous operation, and data-driven analysis.

**Figure 6:**
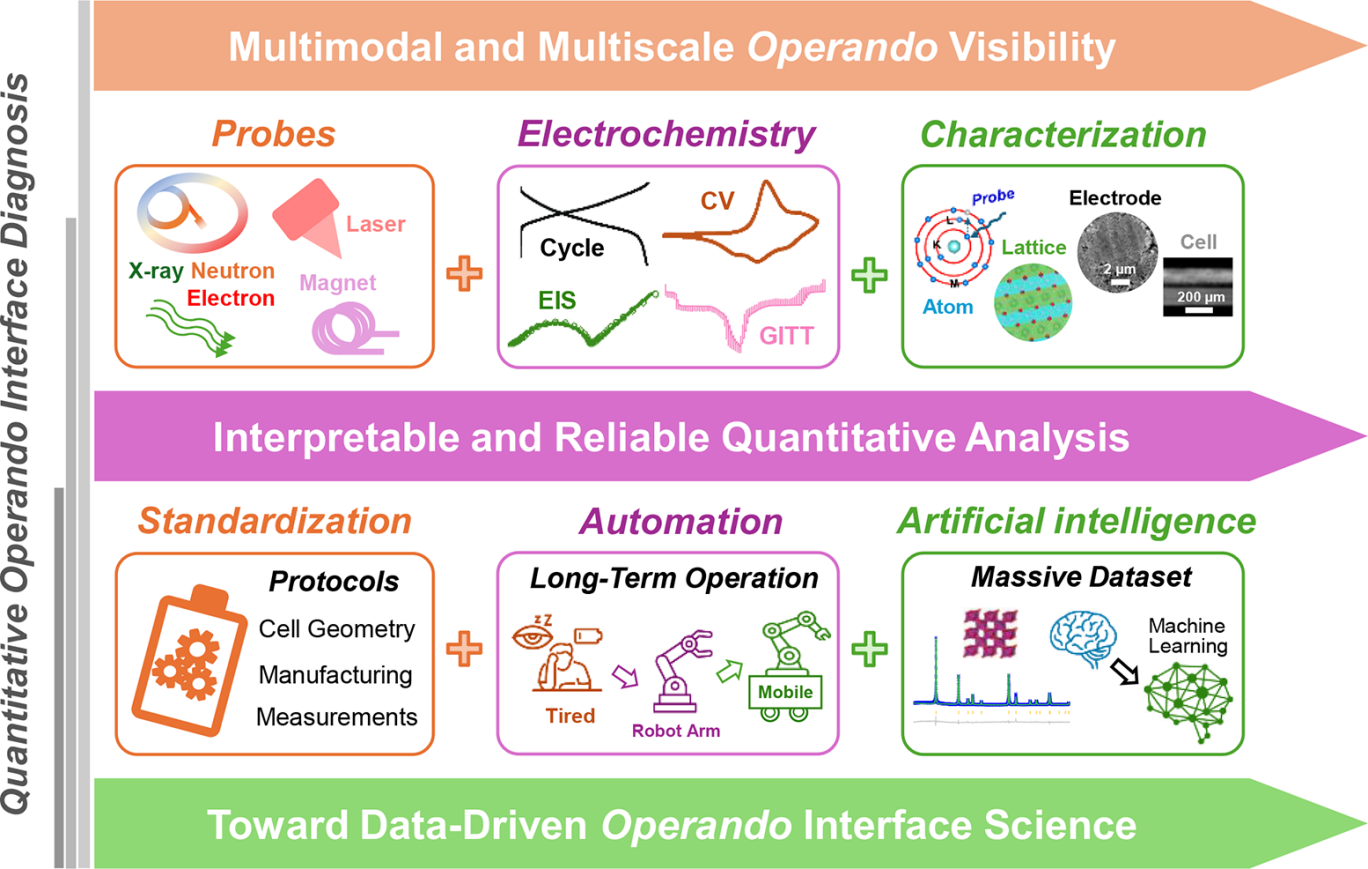
Roadmap enabling reliable quantitative analysis from multimodal *operando* measurements. The upper panel summarizes a multimodal and multiscale *operando* visibility framework that integrates complementary probes, synchronized electrochemical control and structural characterization to track interfacial evolution. The lower panel outlines the capabilities required for interpretable and reliable quantitative analysis, including standardized cell and measurement parameters, autonomous robotic platforms that provide stable long-term experimentation, and artificial intelligence (AI) tools that extract quantitative descriptors from large, multimodal *operando* data sets. Together, these elements establish the experimental and analytical foundations for data-driven *operando* interface science.

Reproducible *operando* measurement begins with standardized sample and cell preparation.^5,29^ Variations in electrode formulation, film thickness, electrolyte volume, wetting, stack pressure, and window or housing materials can significantly influence derived quantities and undermine multimodal comparison. Automated and standardized cell-assembly workflows reduce operator-dependent variability, ensure consistent interfacial environments, and provide reliable baselines for cross-technique and cross-laboratory data sets. Standardization should also explicitly consider beam damage, with radiation dose rate and exposure methodology treated as reportable experimental parameters to ensure meaningful *operando* interpretation.^80^

As different *operando* probes inherently require different cell geometries, reconciliation relies on limiting geometric variability within defined tolerances and using modular, partially interchangeable measurement platforms with shared reference conditions. These approaches help maintain cross-technique consistency and allow data sets collected with complementary probes to be placed on a common quantitative basis. Emerging versatile *operando* cells that support multiple complementary techniques within a shared geometric framework reduce discrepancies between probes and samples by allowing several measurements to be performed on the same cell. These multifunctional designs represent a promising direction for unified multimodal *operando* analysis.^60,62,78,87^

Reliable quantitative interpretation also depends on the long-term measurement stability. Many *operando* experiments require continuous operation over tens of hours or longer, during which alignment, thermal conditions, and electrochemical precision must be maintained. Manual intervention introduces variability and fatigue. Autonomous platforms enable reproducible sample handling, continuous cycling, and uninterrupted monitoring. Emerging mobile robotic systems may further support automated sample exchange and adaptive experimentation guided by real-time data.^88^

AI and machine learning (ML) are increasingly essential for analyzing large and complex data sets produced by multimodal *operando* measurements. Continuous diffraction profiles, spectral sequences, imaging data sets, and electrochemical traces evolve across multiple scales, making manual interpretation impractical and rendering small or emergent signals difficult to detect.^46^ Pattern recognition in ML can automatically identify subtle peak shifts, spectral feature changes, imaging-based structural or morphological evolution, and early-stage anomalies that are easily missed in high-dimensional *operando* data sets. When guided by physical constraints, AI tools can organize heterogeneous data sets, identify correlated evolution across probes, and extract quantitative descriptors.^40,83^ Because *operando* measurements capture interfacial evolution under realistic operating conditions without artifacts introduced by *ex situ* preparation, they provide a strong foundation for such a data-driven analysis.

Together, these developments establish a practical pathway toward quantitative *operando* interface diagnosis. Standardization ensures cross-study comparability, autonomous platforms provide stable long-term measurement, and data-driven analysis expands both the scale and the fidelity of interpretation. The roadmap in Figure 6 operationalizes the conceptual framework introduced in Section 4.2 and enables the transition from *operando* visibility to quantitative and predictive interface science.

## 5 Conclusions and Outlook

*Operando* methodology has transformed the study of buried interfaces by enabling their evolution to be followed directly under operating conditions rather than inferred from altered postoperation states. The analysis from this perspective shows, however, that visibility alone is insufficient for understanding efficiency, degradation or lifetime. The central task is to relate *operando* signals, shaped by coupled chemical, structural, and mechanical processes, to interfacial quantities that are reproducible, comparable, and mechanistically interpretable.

The framework developed here illustrates how multimodal *operando* measurements, physically informed interface models, and data-driven analysis can be combined to extract such quantities in a consistent way. By integrating these elements, qualitative observations can be converted into quantitative descriptors of buried interface evolution, enabling comparisons across materials and across experimental conditions.

Realizing this roadmap will require concerted efforts across multiple stakeholders. National synchrotron and neutron facilities can play a leading role in developing and disseminating standardized, multimodal *operando* end stations, and data acquisition protocols. In parallel, journal editorial policies can incentivize reproducibility by encouraging detailed reporting of cell geometries, beam parameters, and data processing workflows. Ultimately, the transition from *operando* visibility to quantitative diagnosis is not only a technical challenge, but also a sociological and infrastructural one, requiring a more open and collaborative culture in interface science.

Although this perspective focuses on batteries, the concepts are broadly relevant to systems in which electrochemical reactions, mechanical response, and transport interact within buried or confined regions. As *operando* measurements, interface modeling, and data-driven analysis continue to advance, buried interfaces will become quantitatively defined elements of device function, supporting the development of reliable and durable electrochemical technologies across diverse energy materials.
